# Predictors of Post-Thyroidectomy Cervical Hematoma: Does Operative Start Time Matter? A Retrospective Single-Center Study

**DOI:** 10.3390/medicina62071331

**Published:** 2026-07-10

**Authors:** Ufuk Karabacak, Hamdi Burak Piyade, Murat Derebey, Ayfer Kamali Polat, Mahmut Basoglu

**Affiliations:** Department of General Surgery, Faculty of Medicine, Ondokuz Mayıs University, Samsun 55200, Türkiye

**Keywords:** post-thyroidectomy cervical hematoma, thyroidectomy, postoperative bleeding, operative start time, operating room workflow, after-hours surgery

## Abstract

*Background/Objectives*: Post-thyroidectomy cervical hematoma (PCH) is a rare but life-threatening complication that may require emergency airway management and reoperation. Although many patient-, disease-, and surgery-related risk factors have been investigated, the effect of operative scheduling-related variables on PCH remains unclear. This study aimed to evaluate the association between operative start time, operating room case order, surgeon-specific daily case volume, and PCH. *Materials and Methods*: Adult patients who underwent thyroidectomy at our clinic between January 2005 and March 2026 were retrospectively reviewed. Patients requiring reoperation for PCH constituted the case group. All patients operated on by the same surgical team within the same week as each PCH case and who did not develop PCH were included as controls. This approach was used to reduce heterogeneity related to the long study period, institutional workflow, and team rotation in a tertiary teaching hospital. *Results*: A total of 35 patients with PCH and 143 controls were included in the analysis. The study cohort included 178 adult patients with a mean age of 48.4 ± 14.0 years; 48 patients (27.0%) were male, and 31 patients (17.4%) had two or more comorbidities. Among patients who developed PCH, 20 (57.1%) underwent reoperation within the first 12 h, 10 (28.6%) between 12 and 24 h, and 5 (14.3%) after 24 h. In univariable analysis, older age, male sex, hyperthyroidism, lymphocytic thyroiditis, intrathoracic extension, absence of preoperative malignancy diagnosis/suspicion, and operative start time ≥15:00 were associated with PCH. In multivariable analysis, only hyperthyroidism (OR 4.80; 95% CI 1.65–13.92; *p* = 0.004) and operative start time ≥15:00 (OR 13.11; 95% CI 4.55–37.81; *p* < 0.001) were identified as independent risk factors. *Conclusions*: Thyroidectomies starting at or after 15:00, and likely extending into after-hours periods, may carry an increased risk of PCH, potentially reflecting system-level factors such as operating room workflow, non-surgeon perioperative team continuity, anesthesia handover, and postoperative monitoring conditions. These findings should be interpreted in light of the retrospective single-center design, tertiary teaching-hospital setting, limited number of PCH events, and possible residual confounding. Further validation in larger multicenter studies is warranted.

## 1. Introduction

Thyroidectomy is one of the most frequently performed endocrine surgical procedures in daily surgical practice; however, despite its routine nature, it may be associated with rare but potentially life-threatening complications [1,2]. Among the most feared of these is post-thyroidectomy bleeding, which typically occurs in the early postoperative period and reoperation for bleeding has been reported in approximately 0.53–6.5% of all thyroidectomies [3,4,5,6,7,8,9]. Post-thyroidectomy bleeding encompasses a broad clinical spectrum, ranging from minor bleeding that can be managed conservatively or with simple drainage to post-thyroidectomy cervical hematoma (PCH), which may require urgent intervention and reoperation because of its compressive effects [3]. In severe cases, PCH may result in asphyxia, cardiac arrest, or death due to airway compression and laryngeal edema. Therefore, in patients who develop respiratory distress, bedside wound opening with hematoma evacuation to rapidly relieve airway compromise, followed by emergency reoperation, may be required [3,10].

Early recognition and timely management of PCH depend on identifying patients at increased risk of post-thyroidectomy bleeding. Previous studies have investigated numerous patient- and disease-related variables, including age, sex, body mass index (BMI), comorbidities, antithrombotic drug use, thyroid size, hyperthyroidism, and extent of surgery [1,3,7,8,10,11,12,13]. Some studies have also examined non-patient-related factors such as surgeon effect and hospital volume [7,11,12]. However, the reported risk factors remain heterogeneous across studies, and the role of operative scheduling-related variables—particularly operative start time, operating room case order, and surgeon-specific daily case volume—on PCH has not been sufficiently elucidated. Therefore, in addition to established patient-, disease-, and surgery-related variables, this study aimed to investigate the association between operative start time, operating room case order, surgeon-specific daily case volume, and the risk of PCH.

## 2. Materials and Methods

### 2.1. Study Design and Patient Selection

After approval was obtained from the Ondokuz Mayıs University Clinical Research Ethics Committee (approval no. 2026/123), adult patients who underwent thyroidectomy in the Department of General Surgery between 1 January 2005, and 1 March 2026, were retrospectively reviewed. Patients who required reoperation for postoperative bleeding constituted the PCH group.

When establishing the control group, we considered that our institution is a tertiary referral teaching hospital in which surgical and anesthesia team members rotate, and that changes in surgical equipment, operative techniques, and surgeon experience may have occurred over the long study period. Therefore, to reduce time- and team-related heterogeneity, all patients who were operated on by the same surgical team within the same week as each PCH case and who did not develop PCH were included in the control group; the number of controls was not predetermined.

The inclusion criteria were as follows:

1. Adult patients aged 18 years or older.
2. Patients who underwent open cervical thyroidectomy, including lobectomy, total thyroidectomy, or completion thyroidectomy, with or without central and/or lateral neck dissection.
3. For the PCH group, patients who required reoperation for postoperative cervical hematoma after thyroidectomy.
4. For the control group, patients who did not develop PCH and were operated on by the same surgical team within the same week as each PCH case.

The exclusion criteria were as follows:

1. Patients younger than 18 years.
2. Patients with incomplete or unavailable data for key study variables.
3. Patients who underwent thyroidectomy with major concomitant non-thyroid procedures.
4. Patients who underwent emergency or palliative thyroid/neck surgery for acute airway compromise, locally advanced compressive thyroid malignancies, or unresectable/aggressive tumors such as anaplastic thyroid carcinoma.

### 2.2. Data Collection and Variables

Demographic, clinical, operative, and postoperative data were systematically retrieved from medical records. Demographic and clinical variables included age, sex, comorbidities, antithrombotic drug use, hyperthyroidism, thyroiditis, and intrathoracic extension. Operative variables included the type of surgical procedure (lobectomy, total thyroidectomy, or completion thyroidectomy), central or lateral neck dissection, use of intraoperative nerve monitoring (IONM), drain placement, operative start time, operating room case order, and surgeon-specific daily case volume. Operating room case order was defined as the chronological order of the index thyroidectomy among all procedures performed on the same operating room table on that calendar day, including non-thyroid procedures. Surgeon daily case volume was defined as the total number of all procedures performed by the same senior surgeon on that calendar day, including non-thyroid procedures. Postoperative outcome variables included PCH, postoperative length of stay, intensive care unit admission, need for tracheostomy, transient or permanent hypoparathyroidism, and permanent recurrent laryngeal nerve (RLN) palsy. Calendar year was derived from the operation date and categorized into three predefined calendar periods: 2005–2011, 2012–2018, and 2019–2026.

PCH was defined as postoperative cervical hematoma requiring surgical re-exploration and hematoma evacuation. The diagnosis was primarily clinical, based on findings such as cervical swelling or tension, respiratory distress, dysphagia, stridor, or airway compromise. Imaging was not required for diagnosis and was used only in clinically stable or equivocal cases when deemed necessary. Minor bleeding or cervical collections managed without reoperation were not classified as PCH.

Operative start time was obtained from institutional records and was defined as the actual time at which anesthesia induction was initiated. Cases with unavailable operative start time were considered to have missing operative data and were excluded from the analysis. The cutoff of 15:00 was defined before data analysis and was not selected after inspecting the distribution of PCH events. It was selected based on institutional workflow and clinical practice considerations. At our institution, routine working hours end at 16:00 and elective operations are rarely initiated after that time. However, the 15:00 cutoff was not intended to represent the exact time of shift change alone. Rather, it was used as a pragmatic operative scheduling marker to identify thyroidectomies likely to extend into the after-hours period and to reflect the combined effect of several potential system-level factors associated with late-day surgery, including surgeon and team fatigue or time pressure, changes in perioperative team continuity, possible anesthesia handover, and changes in early postoperative monitoring conditions. Given that thyroidectomy procedures typically last approximately 1–2 h, thyroidectomies starting at or after 15:00 are more likely to continue beyond the end of routine working hours and enter the transition from daytime workflow to after-hours care.

Patients with a postoperative serum parathyroid hormone level below 15 pg/mL who required calcium and/or active vitamin D supplementation were considered to have hypoparathyroidism. Hypoparathyroidism that resolved within 6 months was classified as transient, whereas hypoparathyroidism persisting beyond 6 months was classified as permanent. Hyperthyroidism was defined as a documented preoperative hyperthyroid disease, including Graves’ disease, toxic multinodular goiter, and toxic adenoma. Assessment of RLN palsy was symptom-based. Postoperative laryngeal examination was not performed routinely in all patients but was reserved for patients who developed hoarseness or other symptoms suggestive of vocal cord dysfunction. Therefore, RLN palsy was diagnosed only in symptomatic patients in whom laryngeal examination demonstrated impaired vocal cord motion or paralysis, and subclinical RLN palsy may have been missed.

### 2.3. Statistical Analysis

Statistical analyses were performed using IBM SPSS Statistics version 27.0 (IBM Corp., Armonk, NY, USA). The distribution of continuous variables was assessed using histograms, Q–Q plots, and the Shapiro–Wilk test. Normally distributed data are presented as mean ± standard deviation, whereas non-normally distributed data are presented as median (interquartile range [IQR]). Between-group comparisons were performed using the independent-samples t test or the Mann–Whitney U test, as appropriate.

Categorical variables are presented as numbers and percentages. Comparisons of categorical variables were performed using the Pearson chi-square test or Fisher’s exact test, as appropriate. For variables with three categories, the Fisher–Freeman–Halton exact test with Monte Carlo simulation was used when expected cell counts were low. When the overall comparison of a multi-category variable was significant, post hoc pairwise comparisons were performed with Bonferroni correction.

Univariable and multivariable unconditional logistic regression analyses were performed to identify factors associated with PCH. Given the limited number of PCH events (n = 35), the primary multivariable model was intentionally kept parsimonious to reduce the risk of overfitting and preserve model stability. Variables were not selected solely on the basis of univariable statistical significance. Instead, the primary model included variables that were associated with PCH in univariable analysis and were also considered clinically relevant or previously reported in the literature as potential risk factors for post-thyroidectomy bleeding. The main multivariable model therefore included hyperthyroidism, lymphocytic thyroiditis, intrathoracic extension, and operative start time.

Three additional sensitivity analyses were performed to assess the robustness of the primary findings. In the first expanded unconditional model, variables that were associated with PCH in univariable analysis but were not included in the primary model, namely age, sex, and preoperative malignancy diagnosis/suspicion, were added to the main clinical model. In the second unconditional sensitivity model, variables that were not significant in univariable analysis but could still represent clinically relevant confounders, namely operative duration and calendar period, were included.

Because controls were selected from thyroidectomies performed by the same surgical team and during the same calendar week as the corresponding PCH cases, this control-selection strategy may have introduced a partially matched sampling structure. These criteria were used primarily to reduce temporal, workflow-related, and team-related variability in a tertiary teaching-hospital setting, rather than to create a strict fixed-ratio individual matching design. Therefore, unconditional logistic regression was used as the primary analytical approach to evaluate associations within the overall analyzed case–control sample.

To assess whether the partially matched control-selection strategy influenced the main findings, a conditional logistic regression sensitivity analysis was additionally performed using surgical team/calendar week strata. Given the limited number of PCH events and the need to preserve model stability within the matched strata, this conditional model was restricted to the same parsimonious set of variables used in the primary multivariable model: hyperthyroidism, lymphocytic thyroiditis, intrathoracic extension, and operative start time. The results of all three sensitivity analyses are presented in the Supplementary Materials. Results are reported as odds ratios (ORs) with 95% confidence intervals (CIs). A two-sided *p* value < 0.05 was considered statistically significant.

## 3. Results

**Figure 1:**
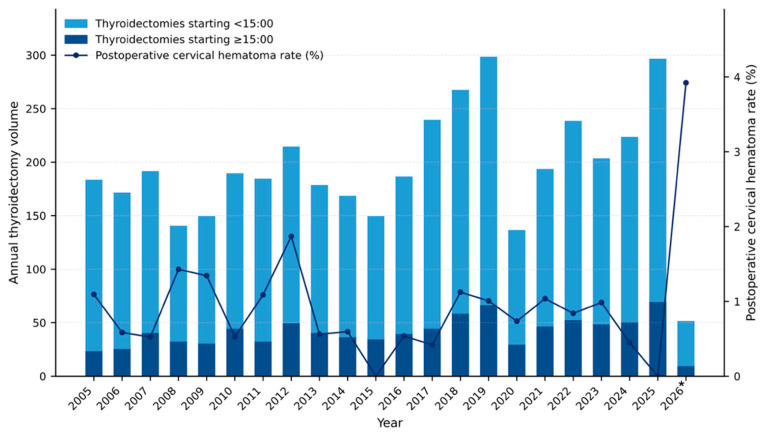
Annual thyroidectomy volume, proportion of thyroidectomies starting before and at/after 15:00, and annual postoperative cervical hematoma rate. * The year 2026 includes cases performed up to 1 March 2026.

**Figure 2:**
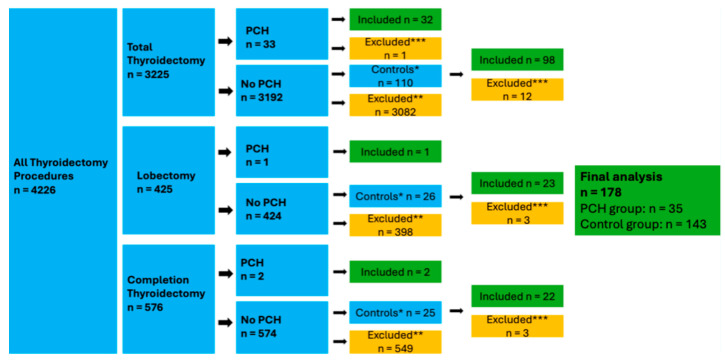
Study flowchart showing patient selection and the inclusion/exclusion process. PCH: post-thyroidectomy cervical hematoma. * Patients operated on by the same surgical team within the same week as each PCH case. ** Patients not eligible as controls because they were not operated on by the same surgical team within the same week as a PCH case. *** Patients excluded because of incomplete or unavailable data. Blue boxes indicate the source population and patient-selection categories, including PCH/no-PCH status and controls; green boxes indicate included patients; yellow boxes indicate excluded or not eligible patients; and arrows indicate patient flow.

A total of 4226 thyroidectomy procedures were performed during the study period. Among these, 36 patients (0.8%) developed PCH requiring reoperation. Annual thyroidectomy volume, the proportion of operations starting at or after 15:00, and the annual PCH rate are shown in Figure 1. One patient in the PCH group was excluded because of unavailable operative start time data. Among patients without PCH, 161 patients were identified as potential controls because they had been operated on by the same surgical team within the same week as a PCH case, whereas 4029 patients were not eligible for control selection according to this predefined control-selection strategy. Among the potential controls, 18 patients were excluded because of incomplete data, including 12 patients with unavailable preoperative clinical data and 6 patients with unavailable operative start time and/or operating room scheduling data. No patient in either the PCH group or the potential control group met the other predefined exclusion criteria. Consequently, 35 patients with PCH and 143 control patients were included in the study, yielding an approximate case-to-control ratio of 1:4 (Figure 2).

**Figure 3:**
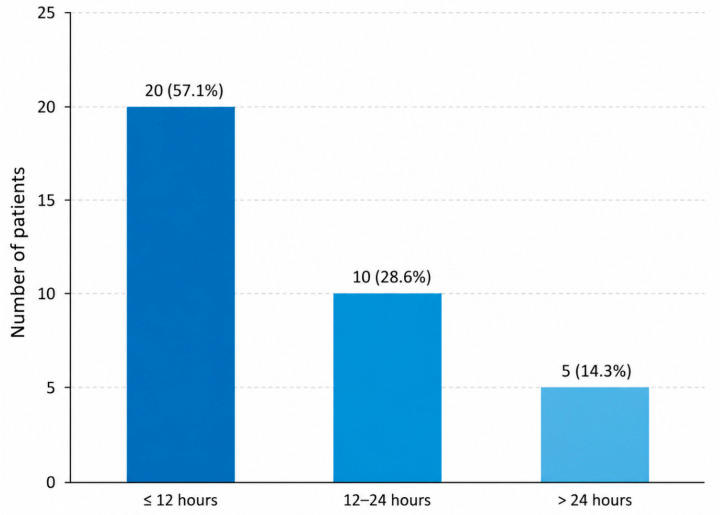
Time to reoperation in patients with post-thyroidectomy cervical hematoma.

Among patients who developed PCH, 20 (57.1%) underwent reoperation within the first 12 h, 10 (28.6%) between 12 and 24 h, and 5 (14.3%) after 24 h (Figure 3). With regard to clinical and demographic characteristics, older age (*p* = 0.019), male sex (*p* = 0.021), hyperthyroidism (*p* < 0.001), thyroiditis (*p* = 0.002), and intrathoracic extension (*p* = 0.002) were significantly associated with PCH. In contrast, PCH was observed less frequently in patients with a preoperative diagnosis or suspicion of malignancy (*p* = 0.002). No significant associations were found between PCH and calendar period, comorbidities, a history of previous thyroid, or neck surgery or antithrombotic drug use (Table 1). Only one patient underwent surgery while continuing acetylsalicylic acid therapy, and this patient developed PCH.

Regarding operative characteristics, the operations in the analytical cohort were performed under the responsibility of 7 senior surgeons, and residents participated in most procedures under senior surgeon supervision; overall, 33 different senior surgeon–resident combinations were identified. The overall distribution of surgical procedures differed significantly between the groups (*p* = 0.024); however, this difference was not maintained in post hoc pairwise comparisons. All five patients who underwent lateral neck dissection also underwent central neck dissection during the same operation. No significant between-group differences were observed with respect to operative duration, central or lateral neck dissection, use of IONM, drain placement, operating room case order, or surgeon-specific daily case volume. In contrast, PCH was significantly more frequent in operations that started at or after 15:00 (*p* < 0.001) (Table 2).

With respect to postoperative outcomes, patients who developed PCH had a longer postoperative hospital stay (*p* < 0.001) and were admitted to the intensive care unit more frequently (*p* < 0.001). No significant between-group differences were observed in the need for tracheostomy, transient hypoparathyroidism, permanent hypoparathyroidism, or permanent recurrent laryngeal nerve palsy (Table 3).

In the univariable logistic regression analysis, age, male sex, hyperthyroidism, thyroiditis, preoperative diagnosis/suspicion of malignancy, intrathoracic extension, and operative start time at or after 15:00 were associated with PCH. In the multivariable analysis, only hyperthyroidism (OR 4.80, 95% CI 1.65–13.92; *p* = 0.004) and operative start time at or after 15:00 (OR 13.11, 95% CI 4.55–37.81; *p* < 0.001) remained independent risk factors (Table 4).

Sensitivity analyses yielded consistent findings. In the expanded clinical model incorporating age, sex, and preoperative malignancy diagnosis/suspicion into the main model, operative start time ≥15:00 (OR 15.56; 95% CI 4.78–50.00; *p* < 0.001) and hyperthyroidism (OR 4.37; 95% CI 1.33–14.29; *p* = 0.015) remained independently associated with PCH, whereas the other covariates did not reach statistical significance. Similarly, in the sensitivity model adjusted for operative duration and calendar period, operative start time ≥15:00 (OR 18.90; 95% CI 5.64–63.28; *p* < 0.001) and hyperthyroidism (OR 4.93; 95% CI 1.64–14.83; *p* = 0.005) remained significant, while operative duration and calendar period were not associated with PCH. In the conditional logistic regression sensitivity analysis using surgical team/calendar week strata, operative start time ≥15:00 (OR 17.73; 95% CI 4.48–70.15; *p* < 0.001) and hyperthyroidism (OR 8.33; 95% CI 2.23–31.11; *p* = 0.002) also remained independently associated with PCH, whereas lymphocytic thyroiditis and intrathoracic extension did not reach statistical significance. These findings support the robustness of the primary results despite the partially matched control-selection strategy. The full results of all three sensitivity analyses are presented in the Supplementary Materials.

## 4. Discussion

The most distinctive finding of our study is that PCH occurred more frequently in thyroidectomies starting at or after 15:00 and extended into after-hours periods. To our knowledge, no previous study has directly examined the relationship between PCH and operative start time in thyroid surgery. The closest study to this concept was conducted by Liu et al., who compared the last operation of the day with other cases without directly analyzing clock time. They found a higher PCH rate in the last-case group in univariable analysis, but this variable was not confirmed as an independent risk factor in multivariable analysis [14]. In contrast, the effect of operative timing on postoperative outcomes has been investigated in other surgical fields. In a meta-analysis including 13 studies from various surgical specialties, Yang et al. showed that after-hours surgery was associated with increased postoperative mortality and morbidity [15]. Similarly, Kelz et al. reported higher postoperative morbidity among non-emergency general and peripheral vascular surgery cases starting after 16:00. They suggested that this association might be explained by system-level factors such as shift change, reduced seniority during later shifts, lower nurse-to-patient ratios, junior resident coverage, and reduced availability of after-hours hospital-wide services [16].

At our institution, regular daytime working hours end at 16:00. In operations continuing beyond this time, the operating surgical team remains unchanged; however, the anesthesiologist, operating room nurses, and operating room support staff are routinely replaced. In addition, the number of staff per patient decreases in both the recovery room and the surgical ward. These changes may contribute to PCH risk by disrupting perioperative team continuity and reducing the intensity of postoperative monitoring. In our cohort, operative duration was numerically shorter among patients who developed PCH, although this difference did not reach statistical significance. In this context, the finding by Canu et al. that shorter operative duration was associated with PCH is noteworthy, although their study did not include data on operative start time or case order [3]. These observations raise the possibility that a tendency to complete procedures more rapidly during after-hours periods may contribute to PCH risk. In addition, the prevention of postoperative complications depends not only on surgical technique and postoperative monitoring but also on continuity of anesthesia care. Jones et al. demonstrated that complete handover of intraoperative anesthesia care in patients undergoing major surgery was associated with increased postoperative complications and mortality [17]. Taken together, these findings suggest that the increased frequency of PCH in thyroidectomies starting at or after 15:00 may be related to multiple interacting system-level factors associated with late-day operative scheduling and transition into after-hours workflow, including surgeon and team fatigue or time pressure, changes in operating room workflow and perioperative team continuity, possible anesthesia handover, and reduced intensity of postoperative monitoring.

Identifying risk factors for PCH is also clinically important in the context of outpatient thyroidectomy, which is increasingly performed in selected patients. Same-day discharge protocols generally rely on careful patient selection, reliable social support, rapid access to emergency medical services, and an adequate postoperative observation period [18]. In some outpatient thyroid surgery protocols, patients are observed for at least 4 h postoperatively, total thyroidectomies are scheduled as the first case of the day, and hemithyroidectomy procedures are planned to be completed by 13:00 [19]. This approach suggests that operative start time, the postoperative observation window, and bleeding risk are considered interrelated factors in daily clinical practice.

Although many studies have investigated risk factors for PCH, the reported results remain heterogeneous. Meta-analyses and large series have identified male sex, older age, hypertension, diabetes mellitus, Graves’ disease, antithrombotic drug use, previous thyroid surgery, high BMI, increased thyroid mass, extent of surgery, neck dissection, low-volume centers, surgeon experience, advanced tumor stage, and lymphocytic thyroiditis as variables associated with PCH; however, the evidence remains inconsistent for many of these factors [1,2,4,5,7,8,11,12,13,14,20,21,22,23,24]. In our study, male sex, older age, hyperthyroidism, absence of preoperative malignancy diagnosis/suspicion, lymphocytic thyroiditis, and operative start time were associated with PCH in the univariable analysis, whereas only hyperthyroidism and operative start time remained independent predictors in the multivariable analysis.

Beyond disease-related factors, anatomical complexity may also influence the perceived risk of postoperative bleeding. Intrathoracic extension is generally considered a marker of increased operative complexity in thyroid surgery. Although sternotomy may occasionally be required, most cases can be completed through a cervical approach [25]. During cervical surgery, externalization of the mediastinal component may require digital dissection in the extracapsular plane, including blind maneuvers [26]. This may raise theoretical concerns regarding hemostatic control and the risk of postoperative cervical hematoma. Nevertheless, despite these concerns, intrathoracic extension was not identified as an independent predictor of PCH in the present study, which is consistent with recent reports showing no independent association between intrathoracic extension and PCH [3,5].

Some studies have reported increased rates of RLN injury and postoperative hypoparathyroidism among patients who develop PCH, attributing these complications both to technical difficulty during the initial operation, with poor delineation of anatomical planes, and to additional tissue trauma during hemostasis at reoperation [5,13]. In contrast, Canu et al. reported a higher rate of RLN injury in the PCH group but, unexpectedly, a lower rate of postoperative hypoparathyroidism. The authors suggested that less aggressive hemostasis might predispose to hematoma while simultaneously better preserving parathyroid vascularization [3]. However, existing studies generally cannot clearly distinguish whether these complications occur during the initial thyroidectomy or during the second exploration. Another clinically important issue related to PCH is the need for tracheostomy, either because of RLN injury or emergency airway management [5,7,27]. In the present study, PCH was not significantly associated with RLN palsy, transient hypoparathyroidism, or permanent hypoparathyroidism (Table 3). The only patient in the PCH group who developed permanent RLN palsy had loss of IONM signal during the initial operation. In the only patient who required tracheostomy, postoperative respiratory distress was the predominant clinical problem; emergency tracheostomy was performed because of difficult intubation during reoperation, and no RLN palsy was detected. As expected, PCH was also associated with an increased postoperative care burden, reflected by higher intensive care unit admission rates and longer hospital stay (Table 3).

Although rare, the most feared consequence of PCH is acute airway compromise caused by cervical compression and laryngeal edema. Therefore, suspected PCH requires early recognition and prompt multidisciplinary management involving the surgical, anesthesia, and nursing teams. In patients with respiratory distress or progressive airway compromise, immediate bedside wound opening and hematoma evacuation may be required before transfer to the operating room. If airway compromise persists despite bedside hematoma evacuation, emergency tracheal intubation should be considered. Because repeated intubation attempts may exacerbate laryngeal edema and further compromise ventilation and oxygenation, tracheal intubation should be performed by an experienced anesthesiologist, with strategies aimed at maximizing first-attempt success, including the use of videolaryngoscopy and a smaller-diameter tracheal tube. If clinically appropriate, awake tracheal intubation may be considered to secure the airway before induction of general anesthesia while preserving spontaneous ventilation. In a cannot-intubate, cannot-oxygenate scenario, early progression to front-of-neck airway access should be considered, with scalpel cricothyroidotomy or emergency tracheostomy [10]. Although the most immediate concern in PCH is airway compromise, its clinical impact may extend beyond acute compression and contribute to broader postoperative morbidity and increased resource utilization.

This study has several limitations. First, PCH is a clinically significant but rare complication; therefore, the number of events was limited despite the long study period. Because of this limited number of events, it was not possible to construct a single, more comprehensive multivariable model including all potentially relevant covariates with sufficient reliability. Second, operative complexity could not be fully captured by the available retrospective data, as there is no single objective criterion that adequately reflects the complexity of thyroidectomy. Although operative duration was numerically shorter among patients who developed PCH and did not differ significantly between groups, duration alone may not fully reflect case complexity. Therefore, the possibility that more complex cases were scheduled later in the day cannot be completely excluded. In addition, some potentially relevant variables, such as BMI and intraoperative blood loss, were not consistently available and may represent unmeasured confounders. Although controls were selected from patients operated on by the same surgical team within the same week and calendar period was not associated with PCH, residual confounding cannot be completely ruled out because of the observational design.

Despite these limitations, this study has important strengths. Operative start time has not been directly investigated in relation to PCH in thyroid surgery, and its association with PCH remained consistent across alternative analytical models. Moreover, operating room case order and surgeon-specific daily case volume were not associated with PCH, suggesting that clock time and transition into after-hours workflow may be more relevant than the numerical sequence of the case or the surgeon’s daily workload. Rather, these findings support the hypothesis that late-day thyroidectomy may reflect the combined effect of system-level factors, including time pressure or fatigue, changes in perioperative team continuity, possible anesthesia handover, and changes in early postoperative monitoring conditions.

## 5. Conclusions

In this study, hyperthyroidism and an operative start time at or after 15:00 were independently associated with PCH after thyroidectomy. The association between late operative start time and PCH suggests that perioperative workflow and postoperative monitoring conditions may be relevant to bleeding risk, beyond patient- and disease-related factors alone. Therefore, in thyroidectomies starting later in the day, meticulous hemostasis, effective team communication, and careful postoperative monitoring may be particularly important. Further larger, multicenter studies are needed to validate these findings.

## Figures and Tables

**Table 1:** Baseline and preoperative characteristics according to post-thyroidectomy cervical hematoma status.

		No PCH n = 143	PCH n = 35	*p* Value
Age (years)	Mean ± SD	47.22 ± 13.76	53.40 ± 14.27	**0.019**
Sex	Male	33 (68.8)	15 (31.3)	**0.021**
	Female	110 (84.6)	20 (15.4)	
Number of comorbidities	<2	122 (83.0)	25 (17.0)	0.079
	≥2	21 (67.7)	10 (32.3)	
Diabetes mellitus	No	124 (80.0)	31 (20.0)	1.000
	Yes	19 (82.6)	4 (17.4)	
Hypertension	No	116 (81.1)	27 (18.9)	0.637
	Yes	27 (77.1)	8 (22.9)	
Cardiovascular disease	No	132 (81.5)	30 (18.5)	0.318
	Yes	11 (68.8)	5 (31.3)	
Pulmonary disease	No	140 (80.0)	35 (20.0)	1.000
	Yes	3 (100.0)	0 (0.0)	
Autoimmune disease	No	138 (80.7)	33 (19.3)	0.625
	Yes	5 (71.4)	2 (28.6)	
Neurological disease	No	140 (80.5)	34 (19.5)	1.000
	Yes	3 (75.0)	1 (25.0)	
Antithrombotic use	No	137 (81.1)	32 (18.9)	0.382
	Yes	6 (66.7)	3 (33.3)	
Previous thyroid or neck surgery	No	113 (78.5)	31 (21.5)	0.198
	Yes	30 (88.2)	4 (11.8)	
Hyperthyroidism	No	128 (85.3)	22 (14.7)	**<0.001**
	Yes	15 (53.6)	13 (46.4)	
Preoperative malignancy diagnosis/suspicion	No	69 (71.9)	27 (28.1)	**0.002**
	Yes	74 (90.2)	8 (9.8)	
Lymphocytic thyroiditis	No	119 (85.6)	20 (14.4)	**0.002**
	Yes	24 (61.5)	15 (38.5)	
Intrathoracic extension	No	126 (85.1)	22 (14.9)	**0.002**
	Yes	17 (56.7)	13 (43.3)	
Calendar period	2005–2011	43 (79.6)	11 (20.4)	0.821
	2012–2018	39 (78.0)	11 (22.0)	
	2019–2026	61 (82.4)	13 (17.6)	

PCH: post-thyroidectomy cervical hematoma. Values are presented as mean ± SD or n (%). Bold *p* values indicate statistical significance at *p* < 0.05.

**Table 2:** Operative characteristics according to post-thyroidectomy cervical hematoma status.

		No PCH n = 143	PCH n = 35	*p* Value
Surgical procedure	Lobectomy	23 (95.8)	1 (4.2)	**0.024**
	Total thyroidectomy	98 (75.4)	32 (24.6)	
	Completion thyroidectomy	22 (91.7)	2 (8.3)	
Central neck dissection	No	124 (79.5)	32 (20.5)	0.575
	Yes	19 (86.4)	3 (13.6)	
Lateral neck dissection	No	139 (80.3)	34 (19.7)	1.000
	Yes	4 (80.0)	1 (20.0)	
Intraoperative nerve monitoring	Hayır	43 (79.6)	11 (20.4)	1.000
	Yes	100 (80.6)	24 (19.4)	
Drain placement	No	10 (100.0)	0 (0.0)	0.214
	Yes	133 (79.2)	35 (20.8)	
Operative start time	<15:00	134 (88.2)	18 (11.8)	**<0.001**
	≥15:00	9 (34.6)	17 (65.4)	
Operating room case order	median (IQR)	2 (1–3)	3 (1–3)	0.820
Operative duration, minutes	median (IQR)	120 (60–120)	80 (60–120)	0.177
Surgeon-specific daily case volume	median (IQR)	3 (2–4)	3 (2–4)	0.933

PCH: post-thyroidectomy cervical hematoma. Values are presented as median (interquartile range, IQR) or n (%). Percentages are sample-based because of the case–control design and do not represent the absolute incidence of PCH in the source population. Bold *p* values indicate statistical significance at *p* < 0.05.

**Table 3:** Postoperative outcomes.

		No PCH n = 143	PCH n = 35	*p* Value
Postoperative length of hospital stay, days	median (IQR)	2.00 (1–3)	4.00 (3–6)	**<0.001**
Intensive care unit admission	No	141 (86.5)	22 (13.5)	**<0.001**
	≤24 h	2 (18.2)	9 (81.8)	
	>24 h	0 (0.0)	4 (100.0)	
Tracheostomy	No	143 (80.8)	34 (19.2)	0.197
	Yes	0 (0.0)	1 (100.0)	
Transient hypoparathyroidism	No	126 (82.4)	27 (17.6)	0.107
	Yes	17 (68.0)	8 (32.0)	
Permanent hypoparathyroidism	No	141 (80.1)	35 (19.9)	1.000
	Yes	2 (100.0)	0 (0.0)	
Permanent recurrent laryngeal nerve palsy	Hayır	143 (80.8)	34 (19.2)	0.197
	Yes	0 (0.0)	1 (100.0)	

PCH: post-thyroidectomy cervical hematoma. Values are presented as median (interquartile range, IQR) or n (%). Bold *p* values indicate statistical significance at *p* < 0.05.

**Table 4:** Univariable and multivariable logistic regression analysis for post-thyroidectomy cervical hematoma.

		Univariable		Multivariable	
		OR (95% CI)	*p* Value	OR (95% CI)	*p* Value
Age (per year increase)		1.03 (1.01–1.06)	**0.021**	—	—
Sex	Male vs. Female	2.50 (1.15–5.42)	**0.020**	—	—
Hyperthyroidism	Yes vs. No	5.04 (2.11–12.03)	**<0.001**	4.80 (1.65–13.92)	**0.004**
Lymphocytic thyroiditis	Yes vs. No	3.72 (1.67–8.28)	**0.001**	2.09 (0.77–5.64)	0.147
Intrathoracic extension	Yes vs. No	4.38 (1.87–10.27)	**0.001**	2.41 (0.81–7.21)	0.115
Operative start time	≥15:00 vs. <15:00	14.06 (5.46–36.21)	**<0.001**	13.11 (4.55–37.81)	**<0.001**
Preoperative malignancy diagnosis/suspicion	Yes vs. No	0.28 (0.12–0.65)	**0.003**	—	—

OR, odds ratio; CI, confidence interval. Bold *p* values indicate statistical significance at *p* < 0.05.

## Data Availability

The datasets used and/or analyzed during the current study are available from the corresponding author on reasonable request.

## Supplementary Materials

The following supporting information can be downloaded at https://www.mdpi.com/article/10.3390/medicina62071331/s1. Table S1: Expanded clinical sensitivity analysis; Table S2: Targeted sensitivity analysis including operative duration and calendar period; Table S3. Conditional logistic regression sensitivity analysis using surgical team/calendar week strata.

## Abbreviations

BMI	Body mass index
CI	Confidence interval
IONM	Intraoperative nerve monitoring
IQR	Interquartile range
OR	Odda ratio
PCH	Post-thyroidectomy cervical hematoma
RLN	Recurrent laryngeal nerve
SD	Standard deviation

## References

[1] Campbell M.J., McCoy K.L., Shen W.T., Carty S.E., Lubitz C.C., Moalem J., Nehs M., Holm T., Greenblatt D.Y., Press D. (2013). A multi-institutional international study of risk factors for hematoma after thyroidectomy. Surgery.

[2] Lee M., Rhee J., Kim Y., Jung Y.H., Ahn S.H., Jeong W.J. (2019). Perioperative risk factors for post-thyroidectomy hematoma: Significance of pain and ketorolac usage. Head Neck.

[3] Canu G.L., Medas F., Cappellacci F., Rossi L., Gjeloshi B., Sessa L., Pennestrì F., Djafarrian R., Mavromati M., Kotsovolis G. (2023). Risk factors for postoperative cervical haematoma in patients undergoing thyroidectomy: A retrospective, multicenter, international analysis (REDHOT study). Front. Surg..

[4] Chiang F., Lin J., Wu C., Lee K., Lu S., Kuo W., Wang L. (2006). Morbidity after total thyroidectomy for benign thyroid disease: Comparison of Graves’ disease and non-Graves’ disease. Kaohsiung J. Med. Sci..

[5] de Carvalho A.Y., Gomes C.C., Chulam T.C., Vartanian J.G., Carvalho G.B., Lira R.B., Kohler H.F., Kowalski L.P. (2021). Risk Factors and Outcomes of Postoperative Neck Hematomas: An Analysis of 5,900 Thyroidectomies Performed at a Cancer Center. Int. Arch. Otorhinolaryngol..

[6] Fassas S., Mamidi I., Lee R., Pasick L., Benito D.A., Thakkar P., Joshi A.S., Goodman J.F. (2021). Postoperative Complications After Thyroidectomy: Time Course and Incidence Before Discharge. J. Surg. Res..

[7] Promberger R., Ott J., Kober F., Koppitsch C., Seemann R., Freissmuth M., Hermann M. (2012). Risk factors for postoperative bleeding after thyroid surgery. Br. J. Surg..

[8] Sun N., Zhang D., Zheng S., Fu L., Li L., Liu S., Li H., Qiu X. (2020). Incidence and Risk Factors of Postoperative Bleeding in Patients Undergoing Total Thyroidectomy. Front. Oncol..

[9] Mulita F., Theofanis G., Verras G.I., Liolis E., Papanikos V., Tchabashvili L., Tasios K., Iliopoulos F., Tsilivigkos C., Kehagias D. (2023). Comparison of postoperative bleeding using harmonic scalpel and LigaSure in thyroid surgery: A 15-year single-centre retrospective study. Med. Glas..

[10] Iliff H.A., El-Boghdadly K., Ahmad I., Davis J., Harris A., Khan S., Lan-Pak-Kee V., O’cOnnor J., Powell L., Rees G. (2022). Management of haematoma after thyroid surgery: Systematic review and multidisciplinary consensus guidelines from the Difficult Airway Society, the British Association of Endocrine and Thyroid Surgeons and the British Association of Otorhinolaryngology, Head and Neck Surgery. Anaesthesia.

[11] Fan C., Zhou X., Su G., Zhou Y., Su J., Luo M., Li H. (2019). Risk factors for neck hematoma requiring surgical re-intervention after thyroidectomy: A systematic review and meta-analysis. BMC Surg..

[12] Jo S.-H., Cho J.-S., Na Y.M., Ryu Y.J., Park M.H., Yoon J.H. (2020). Post-Thyroidectomy Hemorrhage: Time, Place, Risk, and the Surgeon. J. Endocr. Surg..

[13] Mahoney R.C., Vossler J.D., Woodruff S.L., Murayama K.M. (2021). Predictors and Consequences of Hematoma After Thyroidectomy: An American College of Surgeons National Surgical Quality Improvement Program Database Analysis. J. Surg. Res..

[14] Liu J., Li Z., Liu S., Wang X., Xu Z., Tang P. (2016). Risk factors for and occurrence of postoperative cervical hematoma after thyroid surgery: A single-institution study based on 5156 cases from the past 2 years. Head Neck.

[15] Yang N., Elmatite W.M., Elgallad A., Gajdos C., Pourafkari L., Nader N.D. (2019). Patient outcomes related to the daytime versus after-hours surgery: A meta-analysis. J. Clin. Anesth..

[16] Kelz R.R., Hosokawa P., Spitz F., Freeman K.M., Moskowitz M., Henderson W., Itani K. (2008). Time of day is associated with postoperative morbidity: An analysis of the national surgical quality improvement program data. Ann. Surg..

[17] Jones P.M., Cherry R.A., Allen B.N., Jenkyn K.M.B., Shariff S.Z., Flier S., Vogt K.N., Wijeysundera D.N. (2018). Association Between Handover of Anesthesia Care and Adverse Postoperative Outcomes Among Patients Undergoing Major Surgery. JAMA.

[18] Balentine C.J., Sippel R.S. (2016). Outpatient Thyroidectomy: Is it Safe?. Surg. Oncol. Clin. N. Am..

[19] Trottier D.C., Barron P., Moonje V., Tadros S. (2009). Outpatient thyroid surgery: Should patients be discharged on the day of their procedures?. Can. J. Surg..

[20] Perera M., Anabell L., Page D., Harding T., Gnaneswaran N., Chan S. (2016). Risk factors for post-thyroidectomy haematoma. J. Laryngol. Otol..

[21] Saitou M., Akasu H., Jikuzono T., Sen M., Kazusaka H., Matsui M., Sugitani I. (2024). Postoperative Bleeding Risk in Thyroid Surgery: Differences between Conventional and Endoscopic Video-Assisted Neck Surgery Methods. J. Nippon. Med. Sch..

[22] Sarmast Shoushtari M.H., Sherafatmand S., Rostami A., Mohammadi A., Shayesteh B., Farhadi F. (2024). Evaluation of Hematoma Formation after Thyroidectomy Surgery and Its Related Factors. World J. Plast. Surg..

[23] Zhang X., Du W., Fang Q. (2017). Risk factors for postoperative haemorrhage after total thyroidectomy: Clinical results based on 2678 patients. Sci. Rep..

[24] Suzuki S., Yasunaga H., Matsui H., Fushimi K., Saito Y., Yamasoba T. (2016). Factors Associated With Neck Hematoma After Thyroidectomy: A Retrospective Analysis Using a Japanese Inpatient Database. Medicine.

[25] Leivaditis V., Liolis E., Baltayiannis N., Sarof P., Pagoulatou A., Grapatsas K., Antzoulas A., Litsas D., Papadopoulos P.D., Theofanis G. (2025). Diving retrosternal goiter and the dilemma of sternotomy: Indications, predictors and surgical considerations. Pol. J. Thorac. Cardiovasc. Surg..

[26] Battistella E., Pomba L., Sidoti G., Vignotto C., Toniato A. (2022). Retrosternal Goitre: Anatomical Aspects and Technical Notes. Medicina.

[27] Burkey S.H., van Heerden J.A., Thompson G.B., Grant C.S., Schleck C.D., Farley D.R. (2001). Reexploration for symptomatic hematomas after cervical exploration. Surgery.

