# Supplementary material for: Predictors of Post-Thyroidectomy Cervical Hematoma: Does Operative Start Time Matter? A Retrospective Single-Center Study
Source: Medicina (Kaunas). 2026 Jul 10;62(7):1331. doi: 10.3390/medicina62071331 (PMC13413637; doi:10.3390/medicina62071331)
Supplement: Supplementary file 1 [file medicina-62-01331-s001.zip › medicina-4416735-supplementary.pdf]

# Supplementary Logistic Regression Tables

## Supplementary Table S1. Expanded clinical sensitivity analysis

| | | Univariable | | Multivariable | |
|---|---|---|---|---|---|
| | | **OR (95% CI)** | **p value** | **OR (95% CI)** | **p value** |
| **Age (per year increase)** | | **1.03 (**1.01–1.06) | **0.021** | 1.03 (1.00–1.07) | 0.076 |
| **Sex** | Male vs Female | **2.50 (**1.15–5.42) | **0.020** | 2.26 (0.80–6.40) | 0.124 |
| **Hyperthyroidism** | Yes vs No | **5.04 (**2.11–12.03) | **<0.001** | 4.37 (1.33–14.29) | **0.015** |
| **Lymphocytic thyroiditis** | Yes vs No | **3.72 (**1.67–8.28) | **0.001** | 2.86 (0.97–8.47) | 0.058 |
| **Intrathoracic extension** | Yes vs No | **4.38 (**1.87–10.27) | **0.001** | 1.40 (0.44–4.46) | 0.570 |
| **Operative start time** | ≥15:00 vs <15:00 | **14.06 (**5.46–36.21) | **<0.001** | 15.56 (4.78–50.00) | **<0.001** |
| **Preoperative malignancy diagnosis/suspicion** | Yes vs No | **0.28** (0.12–0.65) | **0.003** | 0.35 (0.10–1.18) | 0.091 |

OR, odds ratio; CI, confidence interval.

## Supplementary Table S2. Targeted sensitivity analysis including operative duration and calendar period

| | | Univariable | | Multivariable | |
|---|---|---|---|---|---|
| | | OR (95% CI) | p value | OR (95% CI) | p value |
| **Hyperthyroidism** | Yes vs No | 5.04 (2.11-12.03) | **<0.001** | 4.93 (1.64-14.83) | **0.005** |
| **Lymphocytic thyroiditis** | Yes vs No | 3.72 (1.67-8.28) | **0.001** | 2.16 (0.76-6.09) | 0.146 |
| **Intrathoracic extension** | Yes vs No | 4.38 (1.87-10.27) | **0.001** | 2.89 (0.93-8.96) | 0.067 |
| **Operative start time** | ≥15:00 vs <15:00 | 14.06 (5.46-36.21) | **<0.001** | 18.90 (5.64-63.28) | **<0.001** |
| **Operative duration** | per 10-minute increase | 0.93 (0.84-1.03) | 0.159 | 0.93 (0.82-1.06) | 0.250 |
| **Calendar period, global** | | - | 0.821 | - | 0.173 |
| 2012-2018 vs 2005-2011 | | 1.10 (0.43-2.83) | 0.839 | 1.27 (0.39-4.20) | 0.693 |
| 2019-2026 vs 2005-2011 | | 0.83 (0.34-2.03) | 0.688 | 0.43 (0.12-1.48) | 0.178 |

OR, odds ratio; CI, confidence interval.

## Supplementary Table S3. Conditional logistic regression sensitivity analysis using surgical team/calendar week strata

| | | Multivariable | |
|---|---|---|---|
| | | OR (95% CI) | p value |
| **Hyperthyroidism** | Yes vs No | 8.33 (2.23–31.11) | **0.002** |
| **Lymphocytic thyroiditis** | Yes vs No | 2.73 (0.72–10.35) | 0.139 |
| **Intrathoracic extension** | Yes vs No | 1.91 (0.58–6.28) | 0.288 |
| **Operative start time** | ≥15:00 vs <15:00 | 17.73 (4.48–70.15) | **<0.001** |

OR, odds ratio; CI, confidence interval.
